# Recombinant versus native botulinum neurotoxin type A: structural and pharmacodynamic comparison

**DOI:** 10.1107/S2053230X26009830

**Published:** 2026-10-22

**Authors:** Zongren Yao, Lingyi Xu, Yu Lu, Hui Gong, Xiaoyun Sun, Yaokun Zhao, Jinhuan Chen, Liming Yang, Qingqing Chen, Wu Yang

**Affiliations:** a Chongqing Claruvis Pharmaceutical Co., Ltd, No. 28 Yuefu Avenue, DaDi (International) Life Science Park, A2, Beibei District, Chongqing400700, People’s Republic of China; b WuXi Biologics Biosafety Testing (Shanghai) Co., Ltd, No. 860 Xinyang Road, Lin’gang District, Shanghai Pilot Free Trade, People’s Republic of China; Centro Nacional de Biotecnología – CSIC, Spain

**Keywords:** *Clostridium botulinum*, botulism, botulinum neurotoxin type A, recombinant BoNT/A, cryo-EM, Retoxin

## Abstract

Botulinum neurotoxins (BoNTs) are highly potent exotoxins utilized for esthetic and therapeutic indications. Unlike traditional products derived from *Clostridium botulinum*, this study presents the design, production, structural characterization and preclinical pharmacodynamics of recombinant BoNT type A1 (rBoNT/A, YY001), newly approved by the National Medical Products Administration and the first rBoNT/A product to be licensed for commercialization in the world. Expressed in *Escherichia coli* and purified to greater than 99% homogeneity, YY001 shares a core amino-acid sequence with BoNT/A1 in Botox. The additional five residues at the N-terminus and six residues at the C-terminus of the light chain (LC), as well as two residues at the N-terminus of the heavy chain (HC), originate from residual amino acids after protease cleavage; mass-spectrometric peptide mapping confirmed this sequence fidelity. Cryo-electron microscopy yields a 3.2 Å resolution map of the full-length active rBoNT/A, revealing a canonical LC–H_N_–H_C_ three-domain architecture that predominantly adopts an open conformation, with a root-mean-square deviation of 1.3–1.5 Å relative to native BoNT/A. *In vivo* head-to-head pharmacodynamic studies showed comparable potency between YY001 and Botox in both mouse digit abduction score assay and rat compound muscle action potential assays. Altogether, the structural, biochemical and pharmacological data indicate that YY001 possesses molecular and functional attributes that are highly comparable to those of native BoNT/A, while providing improved manufacturing consistency and reduced biosafety risks.

## Abstract 2

For the first time, the recombinant core botulinum neurotoxin type A (BoNT/A) expressed in *Escherichia coli* and nicked *in vitro* is shown to be structurally identical to native BoNT/A from *Clostridium botulinum*, enabling direct activity comparison between the recombinant product and the native toxin.

## 1. Introduction

Botulinum neurotoxins (BoNTs) are a class of highly neurotoxic proteins produced by *Clostridium botulinum* or related species. The family comprises seven classical serotypes (BoNT/A–BoNT/G) plus the recently identified BoNT/X, while genetic diversity further subdivides these into over 40 distinct subtypes (Peck *et al.*, 2017 ▸; Montal, 2010 ▸; Pirazzini *et al.*, 2017 ▸; Dong *et al.*, 2019 ▸; Rossetto *et al.*, 2014 ▸; Zhang *et al.*, 2017 ▸). Despite their profound toxicity, certain serotypes, most notably BoNT/A1 and BoNT/B1, have been widely adopted in clinical and esthetic medicine (Pirazzini *et al.*, 2017 ▸; Evidente & Adler, 2010 ▸; Dressler, 2012 ▸). A quintessential indication is the treatment of glabellar lines: dynamic facial rhytides that substantially influence expression and perceived age, thereby driving demand for safe and efficacious interventions. The US Food and Drug Administration (FDA) approved BoNT/A for clinical use in 1989 and subsequently authorized its indication for glabellar lines in 2002. In China, BoNT/A products approved for this indication include Botox (Allergan), Dysport (Ipsen), Xeomin (Merz), Letybo (Hugel), Hengli (Lanzhou Biological), Daxxify (Revance) and Hutox (Huons). All currently marketed therapeutic BoNT/A preparations are derived from native toxin proteins purified from *C. botulinum* cultures. However, the pathogenicity of the producing organism and its toxinogenic properties necessitate stringent biosafety safeguards, significantly increasing manufacturing complexity, cost and energy demands.

Compared with the traditional process, recombinant expression in *Escherichia coli* offers a feasible alternative production route, with advantages including reduced biosafety risk, simplified processing and potential cost and energy savings (Weisemann *et al.*, 2015 ▸). Recently, YY001 (rBoNT/A, brand name Retoxin), developed by Chongqing Claruvis Pharmaceutical Co., Ltd, has been approved for marketing and commercialization by the National Medical Products Administration (NMPA) in China, the world’s first approved rBoNT/A, marking a new era in BoNT research and manufacturing. This product was developed by optimizing the gene sequences of the native BoNT/A light chain (LC) and heavy chains (HC) and replacing the native linker between the light and heavy chains with an HRV-3C protease recognition site. During purification, HRV-3C protease is introduced to cleave this site, thereby mimicking natural toxin activation and activating the recombinant toxin from a single polypeptide chain into a disulfide bond-linked dipeptide molecule. Although the coding sequences for both the LC and HC are identical between rBoNT/A and native BoNT/A, the tertiary structure and the protein conformation is more critical for the function of the toxins.

To date, crystallographic and cryo-electron microscopy (cryo-EM) structures of multiple BoNTs (including BoNT/A, BoNT/B, BoNT/E and BoNT/X) have been reported (Gu *et al.*, 2012 ▸; Khanppnavar *et al.*, 2025 ▸; Košenina *et al.*, 2021 ▸; Masuyer *et al.*, 2018 ▸; Swaminathan & Eswaramoorthy, 2000 ▸; Wang *et al.*, 2026 ▸; Lacy *et al.*, 1998 ▸), defining key active sites and functional domains related to toxicity. BoNT family members share a conserved domain architecture: a ∼50 kDa LC, which is a Zn^2+^-dependent endoprotease, and an ∼100 kDa HC, linked by a disulfide bond. The heavy chain can be divided into two domains: the N-terminal translocation domain (H_N_), which mediates LC translocation across the endosomal membrane, and the C-terminal receptor binding domain (H_C_), which is responsible for receptor binding (Košenina *et al.*, 2021 ▸; Lacy *et al.*, 1998 ▸).

Cryo-EM, by capturing proteins in near-native solution conformations, has emerged as the method of choice for resolving full-length botulinum neurotoxin structures (Khanppnavar *et al.*, 2025 ▸; Košenina *et al.*, 2021 ▸). Here, we report the cryo-EM structure of an active rBoNT/A assembled from recombinantly expressed di-chain toxin molecules. The recombinant toxin adopts an overall architecture and preserves key functional motifs that are highly similar to those of native BoNT/A. Mass-spectrometric peptide mapping and preclinical mouse injection studies further confirm that rBoNT/A retains structural integrity and biological potency similar to its native counterpart.

## 2. Materials and methods

### 2.1. Protein expression

rBoNT/A was produced by Claruvis Pharmaceuticals (Chongqing). The gene encoding the amino acids of BoNT/A1 was cloned into pGex vector with a GST tag introduced at the N-terminus. Two peptide linkers containing an HRV-3C protease cleavage site were inserted between the LC and HC and between the GST-tag and LC. The recombinant protein was expressed in *E. coli* BL21 Star (DE3) cells (One Shot, Invitrogen).

The transformed cells were cultured in Terrific Broth (TB; catalogue No. T5574, Merck) within a bioreactor at 37°C. Upon reaching an OD_600_ of 10, the culture was cooled to 18°C, induced with 2 m*M* isopropyl β-d-1-thiogalactopyranoside (IPTG) and harvested after 50 h. Harvested cells were resuspended in lysis buffer (1 *M* KH_2_PO_4_, 100 m*M* NaCl, 2 *M* Na_2_HPO_4_) and lysed via high-pressure homogenization (AH-PILOT2018, ATS) at 1000 bar.

The lysate was clarified by centrifugation (GL-21M, Cence) at 14 000*g* for 45 min. The supernatant was filtered through a 0.45 µm polyethersulfone (PES) capsule filter (catalogue No. KMP8G003HH1, Merck) and then loaded onto an in-house-packed glutathione affinity column (200 ml bed volume; height-to-diameter ratio 2:1; catalogue No. HQ030307001L, Huiyan Bio). On-column cleavage was mediated by 3C protease (15 IU per milligram of substrate protein; GenScript) in phosphate-buffered saline (PBS; 154 m*M* NaCl, 1 m*M* KH_2_PO_4_, 5 m*M* Na_2_HPO_4_ pH 7.2) at a flow rate of 0.02 column volumes (CV) per minute for 30 h. The digested solution was mixed with sodium acetate solution (30 m*M* sodium acetate, 75 m*M* NaCl pH 4.5) at a ratio of 1:10(*w*:*w*) and then loaded onto a HiTrap SP Fast Flow column (catalogue No. 17115101, Cytiva) equilibrated with eluent *A* (45 m*M* sodium acetate, 75 m*M* NaCl pH 5.3) for 30 CV. After sample loading, the column was washed with eluent *A* for 50 CV, followed by gradient elution from 0% to 100% eluent *B* (45 m*M* sodium acetate, 1 *M* NaCl pH 5.3) over 60 CV.

Finally, it was loaded onto an in-house-packed Chromdex 200 PG column (1 l bed volume; height-to-diameter ratio 45:2, catalogue No. KAG0084, Bestchrom) equilibrated with PBS. The final purified rBoNT/A drug substance was formulated at 0.3 mg ml^−1^ in PBS supplemented with 0.1% Polysorbate 80 pH 7.2 and stored at below −60°C.

### 2.2. CE–SDS

The capillary electrophoresis–sodium dodecyl sulfate (CE–SDS) purity was evaluated using the Maurice system (Protein Simple). The drug substance was subjected to buffer exchange. Briefly, the drug substance was removed from storage at −60°C and equilibrated to room temperature. 500 µl was then transferred to a centrifugal filter unit and centrifuged (Fresco 21, Thermo Scientific) at 9600*g* for 10 min. Subsequently, 400 µl 20 m*M* phosphate buffer pH 7.2–7.4 was added to the unit, followed by centrifugation at 9600*g* for 10 min. This buffer-exchange step was repeated two additional times. The concentration of the buffer-exchanged protein was determined by measuring the ultraviolet absorbance at 280 nm using a NanoDrop One spectrophotometer (Thermo Scientific). The protein concentration was required to be not less than 0.75 mg ml^−1^.

For the nonreduced CE–SDS sample preparation, 30 µg of the buffer-exchanged protein and 3 µl 1 mol l^−1^ 2-iodoacetamide (IAA, Sigma) were transferred into a 1.5 ml centrifuge tube; sample buffer (Cat. No. R300109, Protein Simple) was added to a total volume of 100 µl and mixed thoroughly. The mixture was centrifuged at 860*g* for 1 min, incubated at the dark-room temperature for 30 min and finally centrifuged at 9600*g* for 5 min to obtain the test sample.

For the reduced CE–SDS sample preparation, 30 µg buffer-exchanged protein and 5 µl 14.3 mol l^−1^ 2-mercaptoethanol (2-ME, Sigma) were transferred into a 1.5 ml centrifuge tube. Sample buffer was added to bring the total volume to 100 µl and the mixture was mixed thoroughly. The mixture was centrifuged at 860*g* for at least 1 min, incubated at 70°C for 15 min and centrifuged again at 9600*g* for 5 min to obtain the test sample.

The nonreduced and reduced test samples were transferred into a 96-well plate, loaded at 6000 V for 20 s and separated at 5750 V for 35 min using a capillary (catalogue No. 090-157, Protein Simple).

### 2.3. SEC-HPLC

The drug substance was equilibrated to room temperature and 50 µl (15 µg) was injected into the HPLC system. Separation was performed on a size-exclusion chromatography column (AdvanceBio SEC, 7.8 × 300 mm, 2.7 µm; Part No. PL1180-5301, Agilent Technologies). The mobile phase consisted of 200 m*M* arginine, 50 m*M* phosphate, 200 m*M* NaCl pH 7.0. It was delivered at a flow rate of 0.5 ml min^−1^. The column temperature was maintained at 25°C, with UV detection performed at 280 nm for 30 min.

### 2.4. Peptide-mapping MS

The drug substance was buffer-exchanged into 20 m*M* phosphate buffer pH 7.2–7.4 and the protein concentration was measured by ultraviolet absorbance at 280 nm, following the same procedures as described in Section 2.2.

Aliquots containing 100 µg buffer-exchanged protein were divided into two tubes and mixed with an equal volume of 8 *M* urea, followed by incubation at 37°C for 2 h. One aliquot was digested with trypsin (Promega) and Lys-C (Wako), with each enzyme added at an enzyme:protein ratio of 1:20(*w*:*w*). The other aliquot was digested with chymotrypsin at an enzyme:protein ratio of 1:10(*w*:*w*). Both digestion reactions were incubated at 37°C for 18 h. Following enzymatic digestion, tris(2-chloroethyl)phosphate (TCEP, Sigma) was added to each reaction to achieve a final concentration of 20 m*M* and incubated at 37°C for 2 h.

The digested peptides were separated on a reversed-phase column (BEH C18, ACQUITY 2.1 × 150 mm, 1.7 µm, catalogue No. 186002353, Waters) using ultrahigh-performance liquid chromatography (Vanquish UHPLC, ThermoFisher Scientific). The solvent system consisted of 0.1%(*v*/*v*) formic acid in water (*A*) and 0.1%(*v*/*v*) formic acid in acetonitrile (*B*). Consecutive linear gradients of *B* from 1% to 40% within 123 min, then from 40% to 50% within 12 min, and finally from 50% to 95% within 5 min were used. The flow rate was 0.3 ml min^−1^, the column temperature was 55°C and the UV detection wavelength was 214 nm. The peptides were then directed to mass spectrometry (Q Exactive H-FX, ThermoFisher Scientific). The analysis duration was 139 min, operated in positive-ion mode (MSE) with a precursor ion-scan range of 300–2000 *m*/*z*. The MS1 and MS2 resolutions were set to 60 000 and 15 000 at *m*/*z* 200, respectively, and data were acquired in data-dependent acquisition (DDA) mode. The raw data were analyzed using the *Biopharma Finder* version 4.0 (ThermoFisher Scientific) software with the following parameters: mass accuracy set to ±20 p.p.m., variable modification including deamidated (N, Q), oxidation (M, W); the maximum number of modifications per peptide value was set to 2, and the minimum confidence value was set to 0.8. Peptides were searched against the theoretical YY001 sequence database.

### 2.5. Cryo-EM structure determination of rBoNT/A

The structure determination of rBoNT/A was performed by WuXi Biologics Biosafety Testing (Shanghai) Co., Ltd. The cryo-EM grids were prepared by applying 3 µl rBoNT/A protein sample to a glow-discharged holey carbon grid (Quantifoil Au R1.2/1.3, 300 mesh) and were blotted for 3 s under 100% humidity at 4°C before being plunged into liquid ethane using a Vitrobot Mark IV (ThermoFisher). Micrographs were acquired on a Titan Krios microscope (ThermoFisher) operated at 300 kV with a K3 Summit direct electron detector (Gatan). The *EPU* software was used for automated data collection according to standard procedures. A calibrated magnification of 105 000× was used for imaging, yielding a pixel size of 0.824 Å on images. The defocus range was set from −0.8 to −1.8 µm. Each micrograph was dose-fractionated to 50 frames under a total dose of ∼50 e^−^ Å^−2^.

Motion correction was performed using *MotionCor*2 (Zheng *et al.*, 2017 ▸), and the contrast transfer function parameters of the micrographs were estimated using *CTFFIND*-4.1 (Rohou & Grigorieff, 2015 ▸). Other steps of image processing were performed using *RELION* (v.4.0.1; Scheres, 2012 ▸). For rBoNT/A, 3 836 864 particles were automatically picked and extracted from the full dataset of 3001 micrographs. The particles extracted with a binning factor of 3 were processed for two rounds of 3D classification using the initial model of native BoNT/A (PDB entry 3bta; Lacy *et al.*, 1998 ▸) as the reference. One of the 3D classes showed good secondary-structural features and its particles were selected and re-extracted to a pixel size of 0.824 Å. After 3D refinement with *C*1 symmetry and particle polishing using *RELION*, the final 3D reconstruction of rBoNT/A from 237 907 particles yielded an EM map with a resolution of 3.2 Å.

*De novo* atomic model building was performed in *Coot* (Emsley *et al.*, 2010 ▸) on the basis of the 3.2 Å resolution density map of rBoNT/A. The rBoNT/A atomic model was built using native BoNT/A (PDB entry 3bta) as a starting template. The amino-acid assignment was achieved on the basis of the clearly defined density for bulky residues (Phe, Trp, Tyr and Arg). The initial EM density map enabled us to construct a rBoNT/A model comprising LC domain residues 7–437 and HC domain residues 5–39, 45–175 and 181–850, covering 97.6% of the full-length protein. The geometry statistics for models were generated using *MolProbity* (Williams *et al.*, 2018 ▸). All figures were prepared using *ChimeraX* (Pettersen *et al.*, 2021 ▸).

### 2.6. Digit abduction score (DAS)

A total of 110 CD-1 mice (male, average body weight ∼35 g) were randomly assigned into 11 groups (ten per group) and DAS measurements were performed before administration followed by the administration of 10 µl YY001, Botox or negative control at the corresponding dosages by a single intramuscular injection (the middle of gastrocnemius at the right hindlimb; Broide *et al.*, 2013 ▸).

Botox (100 U per vial) was reconstituted with 1 ml normal saline to yield a concentration of 100 U ml^−1^ and then further diluted to a final concentration of 10 U ml^−1^. YY001 was diluted from the drug substance, which was 0.3 mg ml^−1^ in PBS with polysorbate as stated in Section 2.1. Serial dilutions of YY001 were performed with each dilution step no more than 100-fold, and the final concentration was adjusted to 10 U ml^−1^ according to the LD_50_ value of the drug substance; the specific activity of rBoNT/A1 is approximately 100 units per nanogram of protein. Both products were further diluted with normal saline containing 0.5% human serum albumin. A matched vehicle control, consisting of normal saline containing 0.5% human serum albumin, was prepared concurrently. Injections were performed using a Hamilton micro syringe fitted with a 30G needle for the 10 µl injection volume.

The exact dosing groups and dose levels were masked by *Study Director*. The DAS measurement was conducted once each at 4, 8, 12 and 24 h post-dose, followed by once every 12 h from 24 to 72 h post-dose, once every 24 h from 72 to 360 h post-dose and once every 72 h from 360 to 720 h post-dose.

### 2.7. Compound muscle action potential (CMAP)

Test articles YY001 (0.1 U per rat) and Botox (0.1 U per rat) were administrated to Sprague–Dawley (SD) rats (*n* = 3 per group, male, body weight = 300 ± 20 g) by a single gastrocnemius injection; neuromuscular electrophysiological activity was then quantitatively measured through CMAP assay to evaluate the intervention effect of the test articles on neuromuscle electrophysiological function (Torii *et al.*, 2014 ▸).

Reconstitution, dilution procedures, diluent composition and details regarding the injection syringe and needle are described in Section 2.6. The final diluted concentrations for both Botox and YY001 were 3, 9, 27, 81 and 120 U ml^−1^.

CMAP signals were acquired using percutaneous needle electrodes. The stimulating needle electrode was inserted at the level of the fourth lumbar vertebra to predominantly stimulate the sciatic nerve. The recording electrode was placed on the belly of the hindlimb gastrocnemius muscle, the reference electrode was positioned on the gastrocnemius tendon and the ground electrode was fixed at the base of the tail. Electrical stimulation was delivered at 30 mA with a pulse duration of 0.1 msec. Stimulation frequency was set to 1 Hz. Signal acquisition parameters were as follow: high-pass filter, 0.1 Hz; the hardware input range of the BioAmp amplifier was set to 50 mV; sampling rate, 40 kHz. Baseline measurements were performed prior to dosing; the post-dose CMAP values were measured on days 1, 6, 13, 27, 42 and 56. The CMAP amplitude recovery ratio was defined as
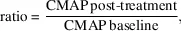
where CMAP_post-treatment_ is the CMAP amplitude after toxin administration and CMAP_baseline_ is the baseline CMAP amplitude prior to treatment.

### 2.8. Potency evaluation (mouse bioassay)

The biological potency of YY001 was quantified using a standardized *in vivo* mouse bioassay (MBA) based on a quantal response design (Pearce *et al.*, 1994 ▸). One unit (1 U) is strictly defined as the median lethal dose (LD_50_) required to induce lethality in 50% of the designated test population via a single intraperitoneal injection under the specific experimental conditions.

Specific-pathogen-free (SPF) CD-1 mice, weighing 18.0–22.0 g, were obtained from Vital River. The animals were acclimated to the institutional vivarium for three days prior to the initiation of dosing. For determination of the LD_50_, the study utilized eight dose cohorts ranging from 0.5 to 2 U per mouse, with each cohort consisting of ten mice. Following the single intraperitoneal injection, the animals were monitored for lethality over a total observation period of 96 h.

The definitive quantal response data (expressed as the ratio of dead to total animals per individual dose cohort) were processed using specialized biostatistical software (for example *SAS Probit* or *GraphPad Prism*). The absolute LD_50_ value for YY001, along with its confidence intervals, was calculated using a standard *Probit* analysis model.

## 3. Results and discussion

### 3.1. Design and characterization of rBoNT/A

rBoNT/A was independently developed and produced by Chongqing Claruvis Pharmaceutical Co., Ltd. The native coding sequences were rationally optimized: the endogenous inter-chain linker was replaced with defined HRV-3C protease recognition motifs, and an N-terminal GST affinity tag, together with its cognate cleavage site, was introduced at the N-terminus to enable the effective purification and subsequent tag removal. The recombinant construct exhibits an overall sequence identity of up to 98.6% relative to the native toxin. Compared with the native toxin, the recombinant construct harbors five additional residues at the LC N-terminus, six residue differences at the LC C-terminus and two residue differences at the HC N-terminus, while key catalytic and functional residues are strictly conserved (Fig. 1 ▸*a* and Supplementary Fig. S1).

After construct assembly, the engineered sequence was expressed in *E. coli* to yield a recombinant precursor protein. Downstream proteolytic processing removed the GST tag and cleaved the engineered inter-chain site to emulate native activation, producing a heterodimeric toxin comprising light and heavy chains that is functionally activated. A multistep chromatographic purification strategy efficiently removed process and product-related impurities, ultimately delivering high-purity rBoNT/A.

The purified material was characterized using orthogonal analytical methods. CE–SDS under nonreducing and reducing conditions was used to assess the molecular composition and electrophoretic profile: nonreducing analysis revealed a predominant intact species at ∼150 kDa, while reducing conditions resolved two principal peaks at ∼100 kDa (HC) and ∼50 kDa (LC), which together accounted for 99.07%, indicating nearly complete cleavage and that the single-chain precursor has been almost entirely converted to the active di-chain form; these results are consistent with native BoNT/A (Fig. 1 ▸*b*). SEC-HPLC quantification indicated a sample purity of 99.49%, further attesting to the efficacy of the purification process (Supplementary Fig. S2). Mass-spectrometric peptide mapping confirmed that the core amino-acid sequence of the recombinant toxin is concordant with that of the designed sequence, supporting sequence integrity and homology (Supplementary Fig. S3). Through sequence refinement, enzymatic activation and rigorous multistep purification, the rBoNT/A produced in this study attains a level of sequence fidelity, molecular composition and purity that is highly comparable to the native toxin, thereby providing a solid platform for subsequent structural and functional evaluation and potential clinical translation.

### 3.2. Structure determination of rBoNT/A

To enable an atomic-level comparison between rBoNT/A and its native counterpart, we determined the structure of full-length rBoNT/A by single-particle cryo-EM. Cryo-EM grids were prepared in vitreous ice and, following extensive data collection and systematic image processing at pH 7.4, we obtained a density map at an overall resolution of 3.2 Å (Fig. 1 ▸*c*, Supplementary Fig. S4 and Table 1 ▸).

The reconstructed map is of high quality: two-dimensional classification readily resolved clear secondary-structure features (Supplementary Fig. S4*a*), permitting unambiguous identification of the catalytic domain of the LC and both the receptor-binding and translocation domains of the HC. The LC density is particularly well defined; aside from several large, intrinsically flexible loop segments that appear to be disordered, the remaining regions are of sufficient clarity for side-chain assignment, covering approximately 96.2% of the LC. For the HC, the N-terminal translocation domain, H_N_, also exhibits well resolved density; conversely, density for the C-terminal receptor-binding domain, H_C_, is comparatively weak. This attenuation likely reflects two contributing factors: intrinsic conformational mobility of the binding domain during receptor engagement and translocation, consistent with previous observations of excessive H_C_ flexibility in apo BoNT/A structures, and a modest degree of preferred particle orientation in our dataset, a phenomenon similarly reported in prior BoNT/B cryo-EM studies (Košenina *et al.*, 2021 ▸; Khanppnavar *et al.*, 2025 ▸). Leveraging available high-resolution H_C_ reference models, we complemented the reconstructed density to complete modeling of H_C_, achieving approximately 98.7% coverage of the heavy chain. Furthermore, due to the considerable flexibility of the protein, the rBoNT/A protein in this study, as well as the BoNT proteins already available in databases, all exhibit validation metrics (including clashscore, Ramachandran outliers and *Q*-score; Table 2 ▸) that are slightly less favorable than those typically observed for structures of comparable resolution. Nonetheless, these limitations do not compromise reliable interpretation of the overall conformation of the toxin and the relative spatial arrangement of its functional domains. The final atomic model together with the corresponding maps have been deposited in the archive, furnishing a rigorous structural framework for subsequent comparative analyses with native BoNT/A and for the delineation of functional sites.

### 3.3. Overall structure of rBoNT/A

rBoNT/A exhibits a structure highly similar to that of native BoNT/A (PDB entry 3bta; Lacy *et al.*, 1998 ▸). It adopts the canonical three-domain architecture: the light chain constitutes the catalytic domain, while the heavy chain comprises the translocation domain and the receptor-binding domain. The translocation domain is particularly distinctive, centered on a pair of elongated α-helices that run along the molecular axis with an axial length of ∼105 Å. In the N-terminal region of this domain a disulfide linkage is formed between Cys7 of the HC and Cys435 of the LC (Fig. 1 ▸*a*), with the two cysteines residing in the heavy and light chains, respectively. Adjacent to this N-terminus, an approximately 50-residue loop or terminal segment wraps around and contacts the periphery of the LC catalytic domain. The disulfide bond and the wrapping segment engage through a complementary interface that interlocks the translocation and catalytic domains, thereby reinforcing HC–LC interactions and ensuring proper assembly of the two chains.

### 3.4. rBoNT/A adopts an open conformation

A summary of published full-length BoNT/A structures is provided in Table 3 ▸. Apart from complexes with the protective factor NTNHA, the toxin principally adopts three global topologies, open, semi-closed and closed, which are broadly interpreted as corresponding to distinct functional and activity states encountered during the biological lifecycle of the toxin. Recent cryo-EM studies indicate that in solution BoNT/A predominantly occupies open and semi-closed conformational states (Khanppnavar *et al.*, 2025 ▸; Wang *et al.*, 2026 ▸).

By direct comparison with representative open and closed reference structures (Fig. 2 ▸), the apo rBoNT/A structure that we have solved adopts an open topology. This assignment rests on a distinct structural hallmark: the absence of direct contact between the LC and the H_C_. Instead, the H_C_ projects to one terminus of the molecule, spatially segregated from the LC, while the H_N_ domain aligns approximately collinearly with the LC along the molecular axis, topologically congruent with the classic open architecture and consistent with crystallographic observations. Notably, an open global arrangement is not unique to BoNT/A; solution studies of full-length BoNT/B have similarly reported a predominantly open population, suggesting that this topology may be a conserved feature across botulinum, with the notable exception of BoNT/E. Several structural studies indicate that BoNT/E preferentially samples a more compact, closed topology (Košenina *et al.*, 2021 ▸; Kumaran *et al.*, 2009 ▸), and this structural divergence may contribute to the faster translocation activity observed for BoNT/E (Wang *et al.*, 2008 ▸).

### 3.5. Comparison of the overall structures of recombinant and native BoNT/A

We next superposed rBoNT/A onto representative open-state BoNT/A structures (Supplementary Fig. S5). The agreement is striking: the global r.m.s.d. is 1.3–1.5 Å over 1257 C^α^ atoms. For context, overlays between cryo-EM and crystallographic models for BoNT/B and BoNT/E yield r.m.s.d.s of ∼1.9 Å (>1218 paired residues) and ∼2.0 Å (>1197 paired residues), respectively, underscoring that the recombinant and native folds coincide to a comparable degree (Košenina *et al.*, 2021 ▸). Because the only available full-length, naturally sourced structure to date (PDB entry 3bta; Lacy *et al.*, 1998 ▸) adopts the open topology, we used it as the reference for subsequent comparisons.

Per-domain comparisons reveal even tighter concordance: LC, H_N_ and H_C_ exhibit r.m.s.d.s of 0.8 Å (431 C^α^), 1.0 Å (411 C^α^) and 1.1 Å (425 C^α^), respectively, each below the global r.m.s.d., indicating that the principal disparities arise from the relative reorientation of rigid domains rather than from substantive perturbations of their internal folds. When superimposing rBoNT/A and native BoNT/A based on the LC domain, compared with native BoNT/A the H_C_ of rBoNT/A is rotated approximately 2° clockwise about an axis perpendicular to the side-view plane, and the H_N_ similarly undergoes a ∼2° clockwise rotation about an axis normal to the top-view plane (Supplementary Fig. S6). Minor local deviations may additionally reflect nonphysiological conformers induced by crystal packing. Pronounced variability in H_C_ position and orientation in our 2D class averages indicates interdomain mobility in solution (Supplementary Fig. S4*a*), consistent with previous reports of alternative apo BoNT/A conformations at neutral pH. In summary, rBoNT/A faithfully reproduces the native backbone architecture while retaining interdomain flexibility that allows it to adopt different conformations in solution.

### 3.6. Comparison of the active sites of recombinant and native BoNT/A

To directly compare the catalytic cores of the recombinant and native toxins, we superimposed their catalytic domains (LC). The domain-level r.m.s.d. is 0.8 Å, and the map resolution permits unambiguous delineation of the four flexible loops that encircle the active site as well as the side-chain conformations of the catalytic residues (Fig. 3 ▸*a* and 3 ▸*b*). The superposition further shows that all secondary-structure elements are preserved and adopt congruent conformations (Figs. 3 ▸*c* and 3 ▸*d*).

It is important to note that LC is a metalloprotease whose activity is sustained by a catalytic Zn^2+^ ion coordinated by the HE*XX*H motif (Fig. 3 ▸*b*). No clear metal-ion density is apparent in our maps. Potential explanations include the absence of supplemental Zn^2+^ during purification, resulting in substoichiometric occupancy or partial metal loss, the inherently limited sensitivity of cryo-EM at this resolution to low-occupancy metals or light atoms, and possible displacement or loss of the metal during vitrification or electron-beam irradiation.

Notwithstanding the absence of visible metal density, the loop architecture and side-chain geometries at the active site are well resolved, supporting a high degree of topological conservation of the catalytic center between the recombinant and native forms. Definitive assignment of metal coordination will require complementary approaches, such as the inclusion of Zn^2+^ during sample preparation, elemental analysis or higher resolution diffraction/imaging data.

### 3.7. Comparative pharmacodynamics of YY001 and Botox

All samples used in pharmacodynamic studies were drug substances. They were removed from storage at below −60°C, equilibrated to room temperature and then formulated for administration. The specific activity in mice was approximately 100 U per nanogram.

To extend our *in vitro* structural and functional studies into an *in vivo* setting and to directly compare the pharmacodynamic profiles of rBoNT/A with those of the naturally derived toxin exemplified by Botox, we performed a systematic head-to-head evaluation using the mouse DAS assay and the rat CMAP model.

The mouse DAS pharmacodynamic study model showed that rBoNT/A inhibited the digit abduction muscle contraction observed at 4 h post-injection, reached its maximum inhibitory effect at 24 h, gradually diminished over time and returned to pre-administration levels at 720 h. Under equivalent dosing conditions, rBoNT/A showed a slightly prolonged recovery compared with Botox. Dose–effect curve analysis revealed no significant difference in the slopes of the dose–response curves between the two drugs; data from the representative 1.2 U dose group are presented below (Fig. 4 ▸).

Direct comparison of nominal unit-based dosing between the two products should be interpreted with caution. This apparent discrepancy may stem from systematic variations in specific activity bio-assay methodologies adopted for different botulinum toxin preparations, along with modest experimental variability introduced during serial dilution of the concentrated drug substance. Since these findings are obtained exclusively from an *in vivo* animal model, extrapolation of the prolonged duration phenotype to human clinical settings cannot be assumed, and prospective clinical investigations will be required to verify whether this pharmacological characteristic is recapitulated in humans.

The rat sciatic nerve extradural stimulation model with local gastrocnemius muscle CMAP pharmacodynamic measurements showed that a single intramuscular injection of 0.33 U kg^−1^ (0.1 U per rat) of YY001 and Botox into the rat gastrocnemius muscle resulted in similar time–CMAP inhibition curves. At the tested concentration, despite transient deviations observed at day 6 and day 13, both drugs exhibited comparable inhibitory effects, which were reversible, returning to pre-administration levels 27 days post-injection. At this concentration, the effects of the two drugs on CMAP (percentage inhibition compared with pre-administration levels) were essentially similar (Fig. 5 ▸). These isolated differences were small and did not translate into a consistent divergence of the overall pharmacodynamic profile in this model.

Taken together, the *in vivo* data indicate broadly comparable pharmacodynamic behavior of rBoNT/A and the naturally derived comparator in the models and conditions tested, with only modest and transient differences observed. These findings should be interpreted with caution owing to model-specific variability, the single-dose design and limited sample sizes; additional studies across multiple doses, time points and models would better define any subtle biological differences.

## 4. Conclusions

In conclusion, YY001 (Retoxin), the first NMPA-approved rBoNT/A produced in *E. coli*, faithfully reproduces the canonical LC–H_N_–H_C_ architecture of native BoNT/A at near-atomic resolution and retains core catalytic and receptor-binding motifs. Orthogonal peptide-mapping mass spectrometry and high-purity biochemical characterization corroborates sequence integrity and product homogeneity, while head-to-head *in vivo* pharmacodynamic assays (DAS and CMAP) demonstrate comparable potency to Botox (Weisemann *et al.*, 2015 ▸). While expressing botulinum neurotoxin type A, *C. botulinum* also expresses multiple accessory proteins that form a complex. However, the precise types and stoichiometric ratios of these proteins remain unclear. Furthermore, the activation of the toxin is mediated by intrinsic enzymes of the bacterium, which are difficult to control (Oguma *et al.*, 1995 ▸; Pitel *et al.*, 2025 ▸). Collectively, these results indicate that recombinant expression can deliver an active, structurally authentic BoNT/A with potential advantages in biosafety, batch consistency and manufacturing scalability (Hasan, 2019 ▸). 

## Supplementary Material

PDB reference: cryo-EM structure of recombinant botulinum neurotoxin A, 24av

EMDB reference: cryo-EM structure of recombinant botulinum neurotoxin A, EMD-69834

Supplementary Figures. DOI: 10.1107/S2053230X26009830/va5072sup1.pdf

## Figures and Tables

**Figure 1:**
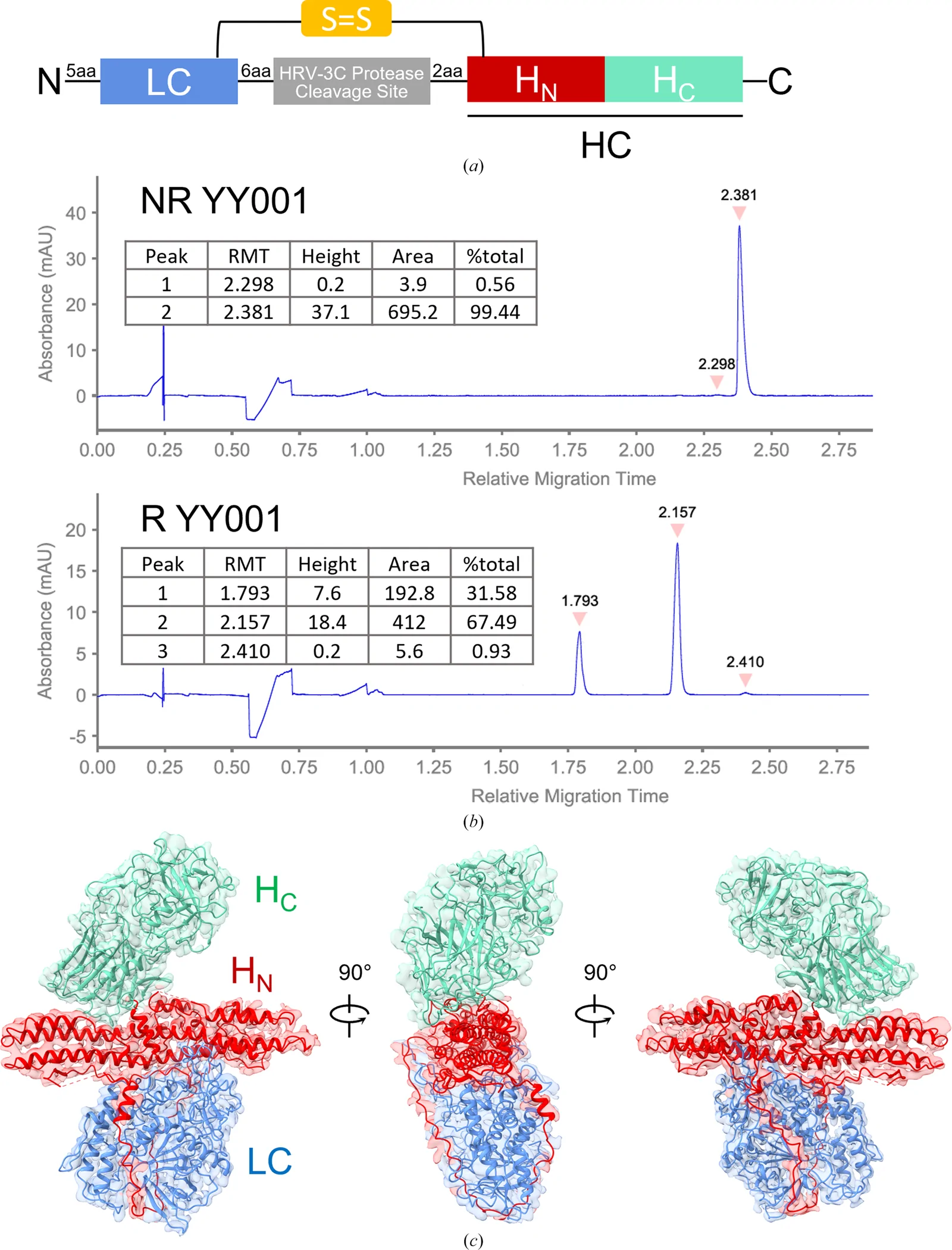
Overall structure of rBoNT/A. (*a*) Schematic depiction of the engineered domain organization of rBoNT/A. (*b*) CE–SDS analysis of rBoNT/A under nonreducing conditions (top) and reducing conditions (bottom). (*c*) Different view of the 3D reconstruction and cartoon representation of rBoNT/A, with each subunit individually colored (LC in blue, H_N_ in red and H_C_ in green).

**Figure 2:**
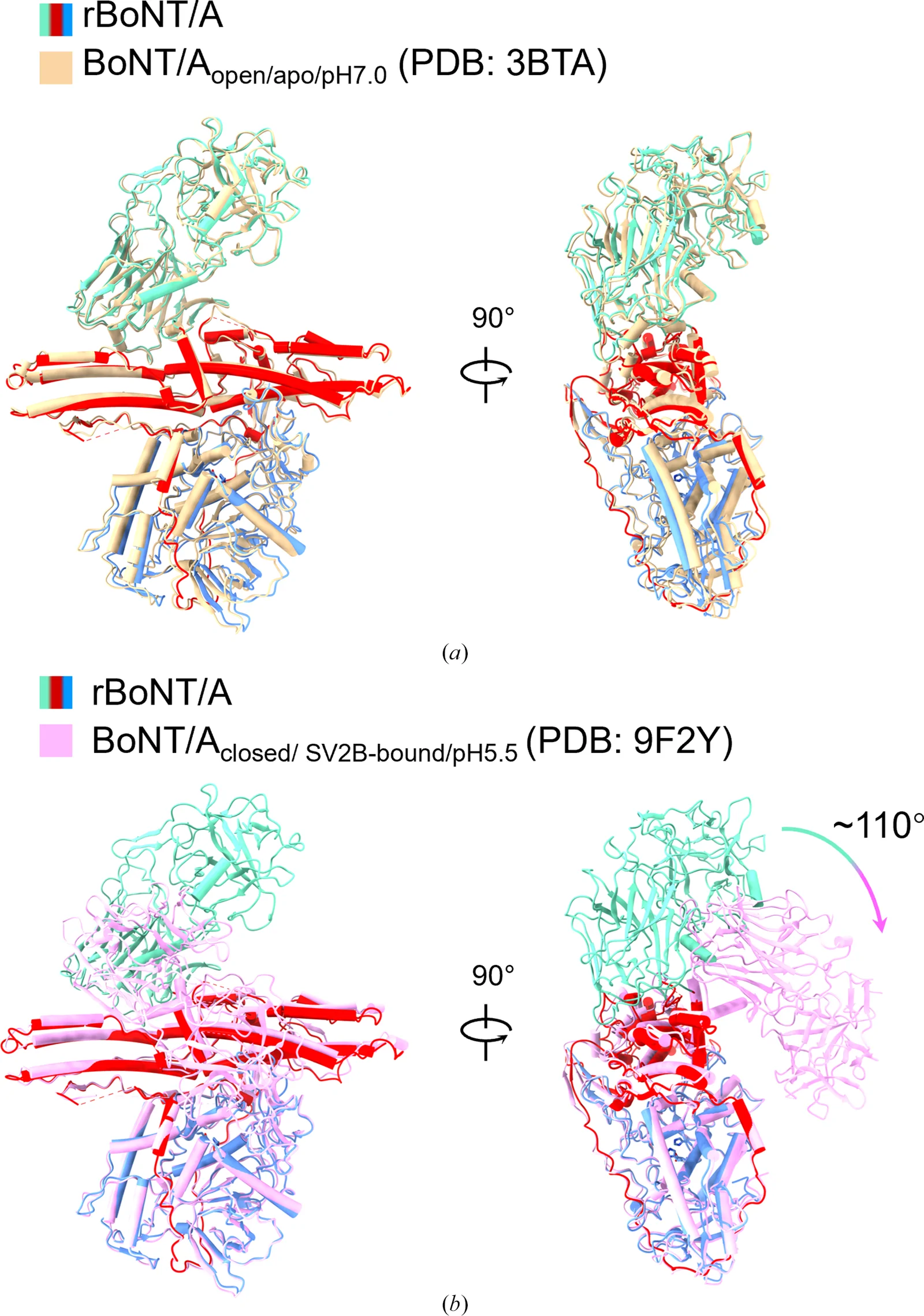
Structural comparison of rBoNT/A with BoNT/A in its open and closed conformations. (*a*) Side view of the superimposition of rBoNT/A (LC in blue, H_N_ in red and H_C_ in green) and BoNT/A_open/apo/pH7.0_ (PDB entry 3bta, wheat) when they are aligned at the LC domain. (*b*) Side view of the superimposition of rBoNT/A (LC in blue, H_N_ in red and H_C_ in green) and BoNT/A_closed/SV2B-bound/pH5.5_ (PDB entry 9f2y, pink) when they are aligned at the LC domain. Green–pink arrows indicate the rotation of the H_C_ domain.

**Figure 3:**
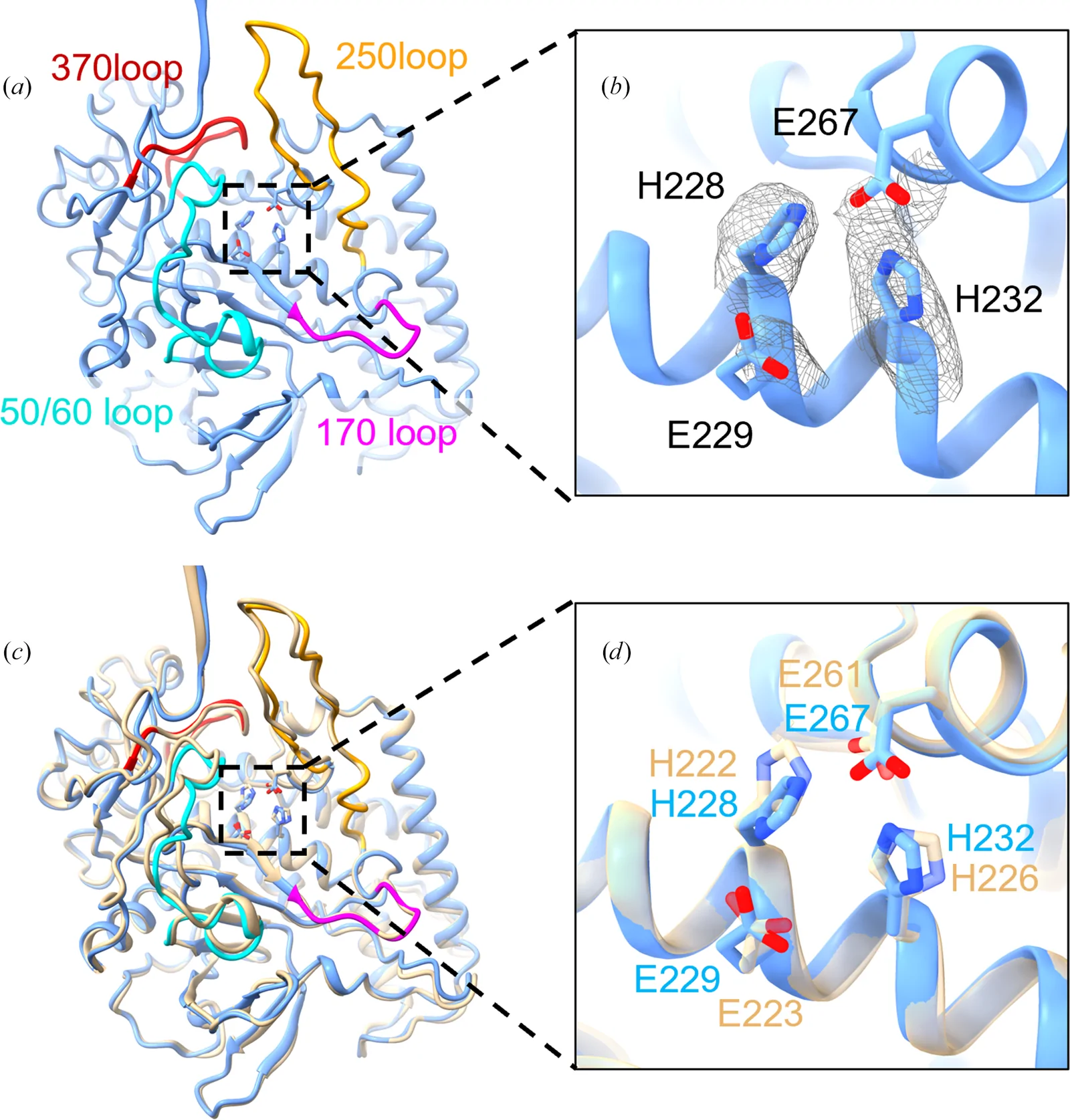
Structural comparison of rBoNT/A with native BoNT/A at the active site. (*a*) Ribbon representation of the rBoNT/A LC structure. The four flexible active-site loops are highlighted in cyan (50/60 loop), magenta (170 loop), orange (250 loop) and red (370 loop). (*b*) Densities of the side chains of His228, Glu229, His232 and Glu267 involved in the network at the Zn^2+^ binding site. (*c*) Side view of the superimposition of LC of rBoNT/A (blue) and native BoNT/A (PDB entry 3bta, wheat) and (*d*) enlarged view.

**Figure 4:**
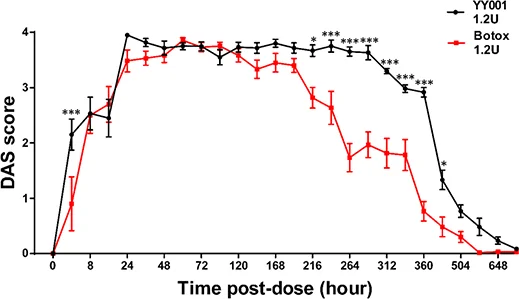
Trend plots of DAS scores of mice following intramuscular injection (the middle of gastrocnemius at the right hindlimb) of YY001 and Botox at 1.2 U and various time points from pre-dose to 720 h post-dose, two-way ANOVA, Bonferroni *post hoc*, *N* = 10.  The DAS scores of the hind limbs digits in the YY001 group at 1.2 U were significantly higher than those in the Botox group at 216, 240, 264, 288, 312, 336, 360 and 432 h post-dose, respectively. Key: *, *p* < 0.05; ***, *p* < 0.001.

**Figure 5:**
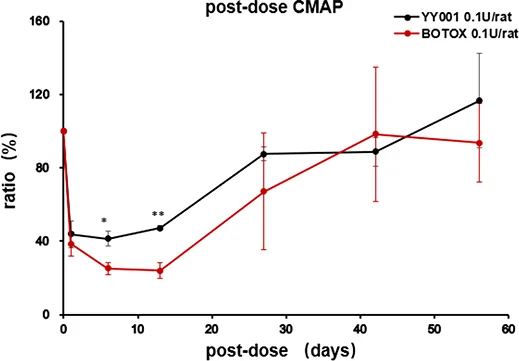
Trend plots of ratio of rat CMAP following intramuscular injection of YY001 and Botox at 0.1 U  per rat. Key: *p, < 0.05; **p, < 0.01.

**Table 1:** Data-collection and refinement statistics for rBoNT/A

Data collection and processing	
Magnification	105000×
Voltage (kV)	300
Electron exposure (e^−^ Å^−2^)	50
Defocus range (µm)	−0.8 to −1.8
Pixel size (Å)	0.824
Symmetry imposed	*C*1
Initial particle images (No.)	3836864
Final particle images (No.)	237907
Map resolution (Å)	3.2
FSC threshold	0.143
Refinement	
Initial model used (PDB code)	3bta
Model resolution (Å)	3.2
Map-sharpening *B* factor (Å^2^)	−80
Model composition	
Non-H atoms	10328
Protein residues	1267
*B* factors (Å^2^)	
Protein	69.82
R.m.s. deviations	
Bond lengths (Å)	0.004
Bond angles (°)	0.639
Validation	
*MolProbity* score	2.19
Clashscore	16.67
Poor rotamers (%)	0
Ramachandran plot	
Favored (%)	92.45
Allowed (%)	6.91
Disallowed (%)	0.64

**Table 2:** Comparison of validation metrics for different BoNT structures

PDB code	Type of BoNT	Resolution (Å)	Method	Clashscore	Ramachandran outliers (%)	*Q*-score
24va (this study)	A	3.2	Cryo-EM	16.67	0.64	0.332
3bta	A	3.2	X-ray	44	6.9	—
3v0a	A	2.7	X-ray	10	0	—
3v0c	A	4.3	X-ray	60	6.9	—
9gkq	A	2.85	Cryo-EM	12	0	0.474
9vcs	A	2.8	Cryo-EM	12	0	0.374
7qfq	B	3.6	Cryo-EM	8	0	0.38
7qfp	E	3.7	Cryo-EM	19	0.6	0.333

**Table 3:** Representative full-length structures of BoNT/A

PDB code	Native	Active	State	pH	Conformation	Method
24va (this study)	No	Yes	Apo	7.4	Open	Cryo-EM
3bta	Yes	Yes	Apo	7.0	Open	X-ray
3v0a	No	No	NTNHA-bound	6.2	Interlock	X-ray
3v0c	No	No	Apo	6.8	Open	X-ray
9f2b	No	No	SV2B-bound	7.4	Open	Cryo-EM
9f2y	No	No	SV2B-bound	5.5	Closed	Cryo-EM
9gkq	No	Yes	Apo	7.4	Open	Cryo-EM
9vcs	No	Yes	Apo	7.4	Semi-closed	Cryo-EM

## Data Availability

Structure coordinates and cryo-EM density maps for rBoNT/A have been deposited in the Protein Data Bank as entry 24va and EMDB as entry EMD-69834.
