# Supplementary material for: Recombinant versus native botulinum neurotoxin type A: structural and pharmacodynamic comparison
Source: Acta Crystallogr F Struct Biol Commun. 2026 Oct 22;82(Pt 11):391–401. doi: 10.1107/S2053230X26009830 (PMC13717837; doi:10.1107/S2053230X26009830)
Supplement: Supplementary file 1 [file f-82-00391-sup1.pdf]

STRUCTURAL BIOLOGY COMMUNICATIONS

Volume 82 (2026)

Supporting information for article:

Recombinant versus native botulinum neurotoxin type A: structural and pharmacodynamic comparison

Zongren Yao, Lingyi Xu, Yu Lu, Hui Gong, Xiaoyun Sun, Yaokun Zhao, Jinhuan Chen, Liming Yang, Qingqing Chen and Wu Yang

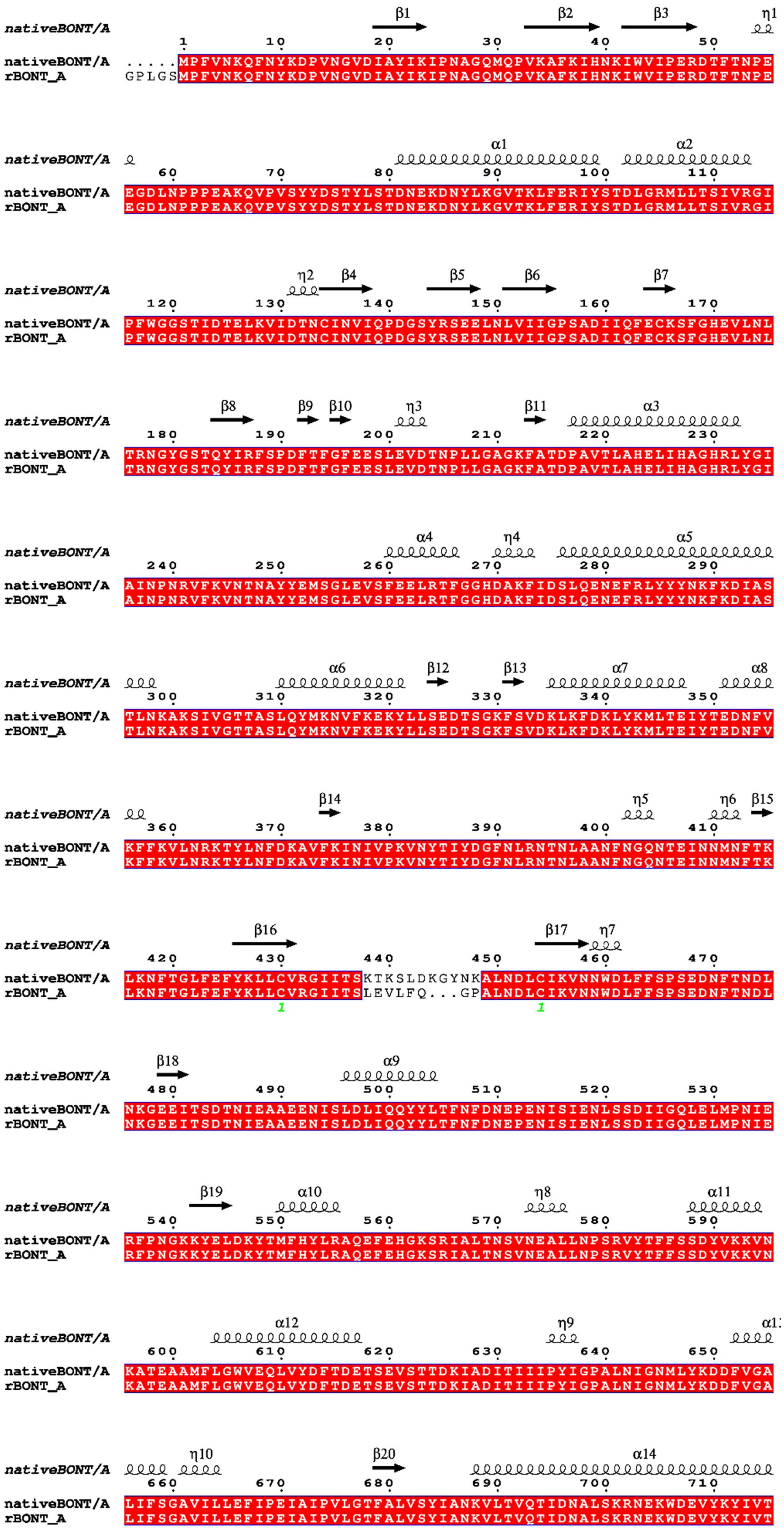

nativeBONT/A β1 β2 β3 η1
nativeBONT/A .....MPFVNKQFNYKDPVNGVDIAYIKIPNAGQMQPVKAFKIHNKIWVIPERDTFTNPE
rBONT_A GPLGSMPFVNKQFNYKDPVNGVDIAYIKIPNAGQMQPVKAFKIHNKIWVIPERDTFTNPE
nativeBONT/A α1 α2
nativeBONT/A EGDLNPPPEAKQVPVSYYDSTYLSTDNEKDNYLKGVTKLFERIYSTDLGRMLLTSIVRGI
rBONT_A EGDLNPPPEAKQVPVSYYDSTYLSTDNEKDNYLKGVTKLFERIYSTDLGRMLLTSIVRGI
nativeBONT/A η2 β4 β5 β6 β7
nativeBONT/A PFWGGSTIDTELKVIDTNCINVIQPDGSYRSEELNLVIIGPSADIIQFECKSFGHEVLNL
rBONT_A PFWGGSTIDTELKVIDTNCINVIQPDGSYRSEELNLVIIGPSADIIQFECKSFGHEVLNL
nativeBONT/A β8 β9 β10 η3 β11 α3
nativeBONT/A TRNGYGSTQYIRFSPDFTFGFEESLEVDTNPLLGAGKFATDPAVTLAHELIHAGHRLYGI
rBONT_A TRNGYGSTQYIRFSPDFTFGFEESLEVDTNPLLGAGKFATDPAVTLAHELIHAGHRLYGI
nativeBONT/A α4 η4 α5
nativeBONT/A AINPNRVFKVNTNAYYEMSGLEVSFEELRTFGGHDAKFIDSLQENEFRLYYYNKFKDIAS
rBONT_A AINPNRVFKVNTNAYYEMSGLEVSFEELRTFGGHDAKFIDSLQENEFRLYYYNKFKDIAS
nativeBONT/A α6 β12 β13 α7 α8
nativeBONT/A TLNKAKSIVGTTASLQYMKNVFKEKYLLSEDTSGKFSVDKLKFDKLYKMLTEIYTEDNFV
rBONT_A TLNKAKSIVGTTASLQYMKNVFKEKYLLSEDTSGKFSVDKLKFDKLYKMLTEIYTEDNFV
nativeBONT/A β14 η5 η6 β15
nativeBONT/A KFFKVLNRKTYLNFDKAVFKINIVPKVNYTIYDGFNLRNTNLAANFNGQNTEINNMNFTK
rBONT_A KFFKVLNRKTYLNFDKAVFKINIVPKVNYTIYDGFNLRNTNLAANFNGQNTEINNMNFTK
nativeBONT/A β16 β17 η7
nativeBONT/A LKNFTGLFEFYKLLCVRGIITSKTKSLDKGYNKALNDLCIKVNNWDLFFSPSEDNFTNDL
rBONT_A LKNFTGLFEFYKLLCVRGIITSLEVLFQ...GPALNDLCIKVNNWDLFFSPSEDNFTNDL
nativeBONT/A β18 α9
nativeBONT/A NKGEEITSDTNIEAAEENISLDLIQQYYLTFNFDNEPENISIENLSSDIIGQLELMPNIE
rBONT_A NKGEEITSDTNIEAAEENISLDLIQQYYLTFNFDNEPENISIENLSSDIIGQLELMPNIE
nativeBONT/A β19 α10 η8 α11
nativeBONT/A RFPNGKKYELDKYTMFHYLRAQEFEHGKSRIALTNSVNEALLNPSRVYTFFSSDYVKKVN
rBONT_A RFPNGKKYELDKYTMFHYLRAQEFEHGKSRIALTNSVNEALLNPSRVYTFFSSDYVKKVN
nativeBONT/A α12 η9
nativeBONT/A KATEAAMFLGWVEQLVYDFTDETSEVSTTDKIADITIIIPYIGPALNIGNMLYKDDFVGA
rBONT_A KATEAAMFLGWVEQLVYDFTDETSEVSTTDKIADITIIIPYIGPALNIGNMLYKDDFVGA
nativeBONT/A η10 β20 α14
nativeBONT/A LIFSGAVILLEFIPEIAIPVLGTFALVSYIANKVLTVQTIDNALSKRNEKWDEVYKYIVT
rBONT_A LIFSGAVILLEFIPEIAIPVLGTFALVSYIANKVLTVQTIDNALSKRNEKWDEVYKYIVT

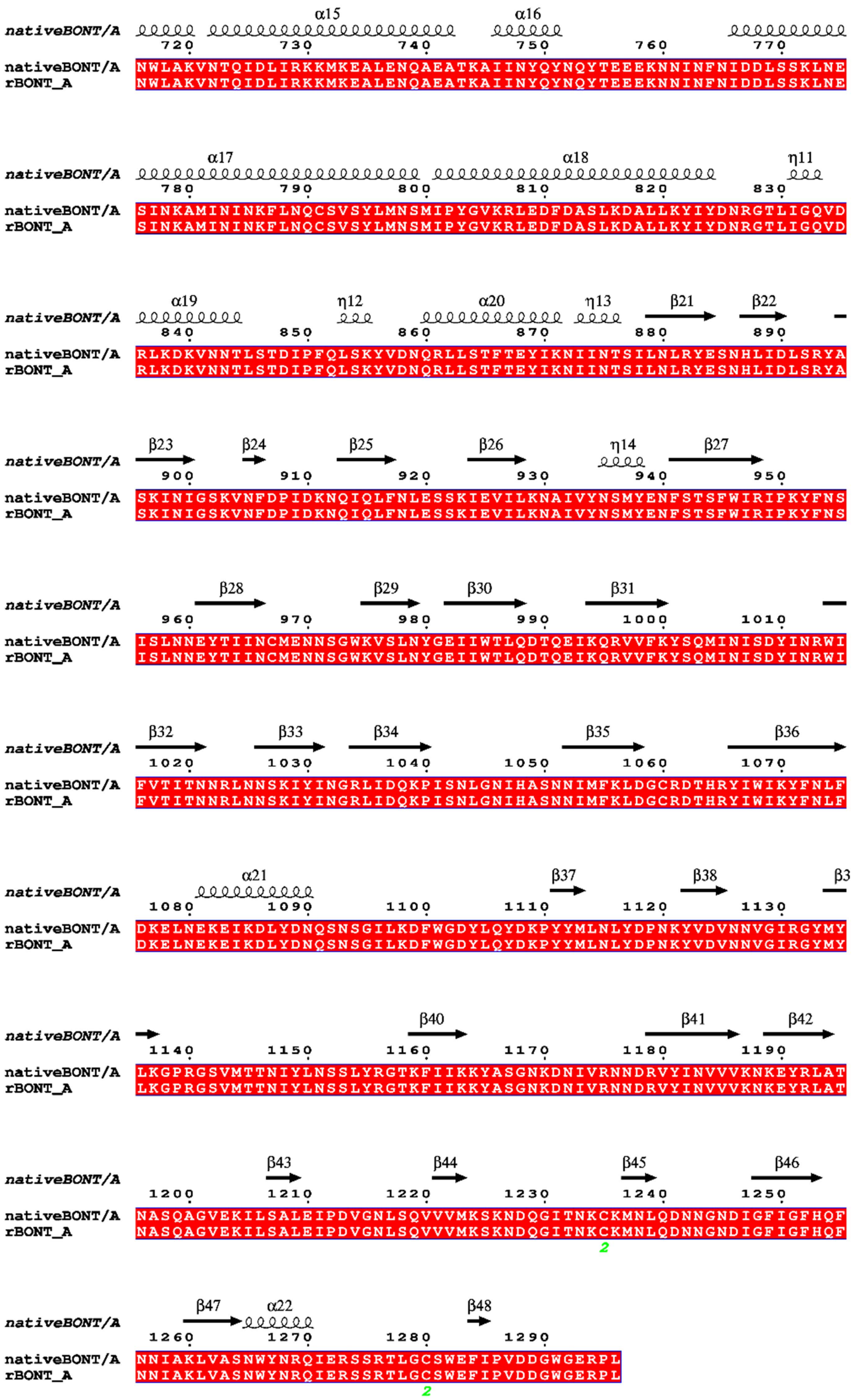

Figure S1 Sequence alignment of rBoNT/A and native BoNT/A. Sequences are aligned with ClustalW and presented by ENDscript. Secondary structural elements of BoNT/A are indicated above the sequence alignment. Accession number of native BoNT/A is WP_011948511.1.

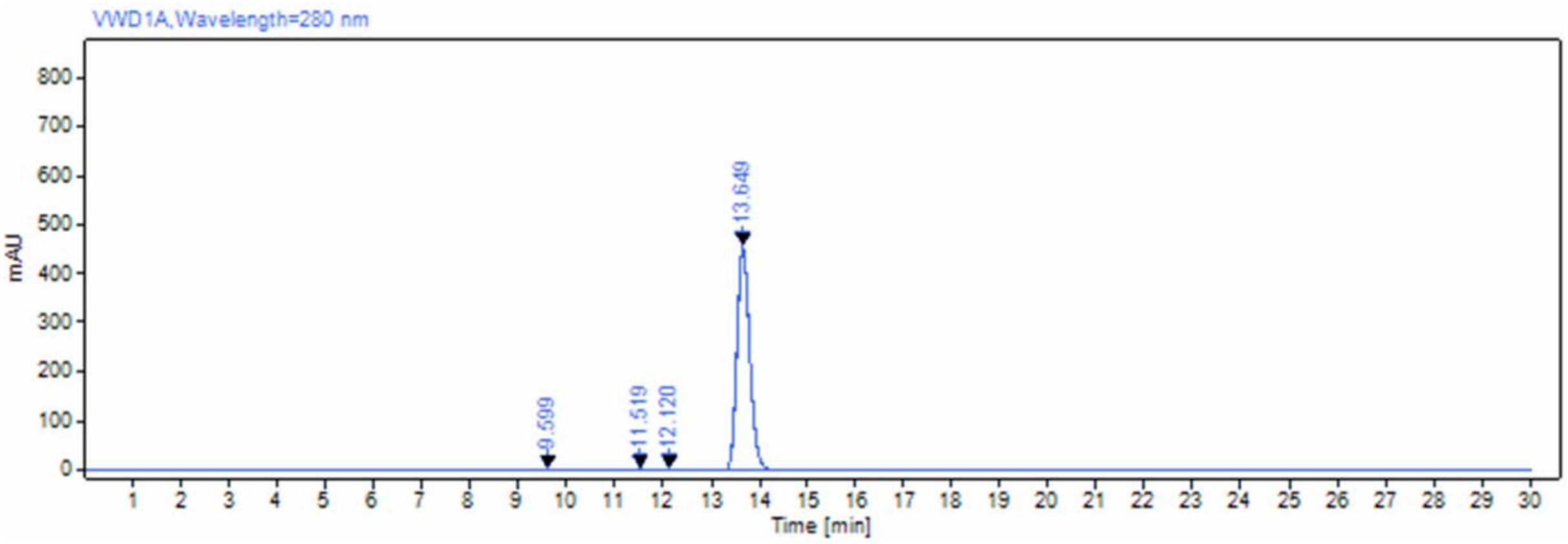

Peak	RT (min)	Height	Area	Area%
1	9.599	0.03	0.83	0.01
2	11.519	0.7	16.47	0.19
3	12.12	0.63	26.28	0.31
4	13.649	455.55	8444.77	99.49

Figure S2 SEC-HPLC analysis of rBoNT/A.

a

LC

1 to 80	GPLGSMPFVN	KQFNYKDPVN	GVDIAYIKIP	NAGQMQPVKA	FKIHNKIWVI	PERDTFTNPE	EGDLNPPPEA	KQVPVSYYDS
81 to 160	TYLSTDNEKD	NYLKGVTKLF	ERIYSTDLGR	MLLTSIVRGI	PFWGGSTIDT	ELKVIDTNCI	NVIQPDGSYR	SEELNLVIIG
161 to 240	PSADIIQFEC	KSFGHEVLNL	TRNGYGSTQY	IRFSPDFTFG	FEESLEVDTN	PLLGAGKFAT	DPAVTLAHEL	IHAGHRLYGI
241 to 320	AINPNRVFKV	NTNAYYEMSG	LEVSFEELRT	FGGHDAKFID	SLQENEFRLY	YYNKFKDIAS	TLNKAKSIVG	TTASLQYMKN
321 to 400	VFKEKYLLSE	DTSGKFSVDK	LKFDKLYKML	TEIYTEDNFV	KFFKVLNRKT	YLNFDKAVFK	INIVPKVNYT	IYDGFNLRNT
401 to 448	NLAANFNGQN	TEINNMNFTK	LKNFTGLFEF	YKLLCVRGII	TSLEVLFQ			

HC

1 to 80	GPALNDLCIK	VNNWDLFFSP	SEDNFTNDLN	KGEEITSDTN	IEAAEENISL	DLIQQYYLTF	NFDNEPENIS	IENLSSDIIG
81 to 160	QLELMPNIER	FPNGKKYELD	KYTMFHYLRA	QEFEHGKSRI	ALTNSVNEAL	LNPSRVYTFF	SSDYVKKVNK	ATEAAMFLGW
161 to 240	VEQLVYDFTD	ETSEVSTTDK	IADITIIIPY	IGPALNIGNM	LYKDDFVGAL	IFSGAVILLE	FIPEIAIPVL	GTFALVSYIA
241 to 320	NKVLTVQTID	NALSKRNEKW	DEVYKYIVTN	WLAKVNTQID	LIRKKMKEAL	ENQAEATKAI	INYQYNQYTE	EEKNNINFNI
321 to 400	DDLSSKLNES	INKAMININK	FLNQCSVSYL	MNSMIPYGVK	RLEDFDASLK	DALLKYIYDN	RGTLIGQVDR	LKDKVNNTLS
401 to 480	TDIPFQLSKY	VDNQRLLSTF	TEYIKNIINT	SILNLRYESN	HLIDLSRYAS	KINIGSKVNF	DPIDKNQIQL	FNLESSKIEV
481 to 560	ILKNAIVYNS	MYENFSTSFW	IRIPKYFNSI	SLNNEYTIIN	CMENNSGWKV	SLNYGEIIWT	LQDTQEIKQR	VVFKYSQMIN
561 to 640	ISDYINRWIF	VTITNNRLNN	SKIYINGRLI	DQKPISNLGN	IHASNNIMFK	LDGCRDTHRY	IWIKYFNLFD	KELNEKEIKD
641 to 720	LYDNQSNSGI	LKDFWGDYLQ	YDKPYYMLNL	YDPNKYVDVN	NVGIRGYMYL	KGPRGSVMTT	NIYLNSSLYR	GTKFIIKKYA
721 to 800	SGNKDNIVRN	NDRVYINVVV	KNKEYRLATN	ASQAGVEKIL	SALEIPDVGN	LSQVVVMKSK	NDQGITNKCK	MNLQDNNGND
801 to 850	IGFIGFHQFN	NIAKLVASNW	YNRQIERSSR	TLGCSWEFIP	VDDGWGERPL			

b

LC

1 to 80	GPLGSMPFVN	KQFNYKDPVN	GVDIAYIKIP	NAGQMQPVKA	FKIHNKIWVI	PERDTFTNPE	EGDLNPPPEA	KQVPVSYYDS
81 to 160	TYLSTDNEKD	NYLKGVTKLF	ERIYSTDLGR	MLLTSIVRGI	PFWGGSTIDT	ELKVIDTNCI	NVIQPDGSYR	SEELNLVIIG
161 to 240	PSADIIQFEC	KSFGHEVLNL	TRNGYGSTQY	IRFSPDFTFG	FEESLEVDTN	PLLGAGKFAT	DPAVTLAHEL	IHAGHRLYGI
241 to 320	AINPNRVFKV	NTNAYYEMSG	LEVSFEELRT	FGGHDAKFID	SLQENEFRLY	YYNKFKDIAS	TLNKAKSIVG	TTASLQYMKN
321 to 400	VFKEKYLLSE	DTSGKFSVDK	LKFDKLYKML	TEIYTEDNFV	KFFKVLNRKT	YLNFDKAVFK	INIVPKVNYT	IYDGFNLRNT
401 to 448	NLAANFNGQN	TEINNMNFTK	LKNFTGLFEF	YKLLCVRGII	TSLEVLFQ			

HC

1 to 80	GPALNDLCIK	VNNWDLFFSP	SEDNFTNDLN	KGEEITSDTN	IEAAEENISL	DLIQQYYLTF	NFDNEPENIS	IENLSSDIIG
81 to 160	QLELMPNIER	FPNGKKYELD	KYTMFHYLRA	QEFEHGKSRI	ALTNSVNEAL	LNPSRVYTFF	SSDYVKKVNK	ATEAAMFLGW
161 to 240	VEQLVYDFTD	ETSEVSTTDK	IADITIIIPY	IGPALNIGNM	LYKDDFVGAL	IFSGAVILLE	FIPEIAIPVL	GTFALVSYIA
241 to 320	NKVLTVQTID	NALSKRNEKW	DEVYKYIVTN	WLAKVNTQID	LIRKKMKEAL	ENQAEATKAI	INYQYNQYTE	EEKNNINFNI
321 to 400	DDLSSKLNES	INKAMININK	FLNQCSVSYL	MNSMIPYGVK	RLEDFDASLK	DALLKYIYDN	RGTLIGQVDR	LKDKVNNTLS
401 to 480	TDIPFQLSKY	VDNQRLLSTF	TEYIKNIINT	SILNLRYESN	HLIDLSRYAS	KINIGSKVNF	DPIDKNQIQL	FNLESSKIEV
481 to 560	ILKNAIVYNS	MYENFSTSFW	IRIPKYFNSI	SLNNEYTIIN	CMENNSGWKV	SLNYGEIIWT	LQDTQEIKQR	VVFKYSQMIN
561 to 640	ISDYINRWIF	VTITNNRLNN	SKIYINGRLI	DQKPISNLGN	IHASNNIMFK	LDGCRDTHRY	IWIKYFNLFD	KELNEKEIKD
641 to 720	LYDNQSNSGI	LKDFWGDYLQ	YDKPYYMLNL	YDPNKYVDVN	NVGIRGYMYL	KGPRGSVMTT	NIYLNSSLYR	GTKFIIKKYA
721 to 800	SGNKDNIVRN	NDRVYINVVV	KNKEYRLATN	ASQAGVEKIL	SALEIPDVGN	LSQVVVMKSK	NDQGITNKCK	MNLQDNNGND
801 to 850	IGFIGFHQFN	NIAKLVASNW	YNRQIERSSR	TLGCSWEFIP	VDDGWGERPL			

Figure S3 Mass spectrometric analysis of rBoNT/A. (a) Peptide coverage following chymotrypsin digestion. (b) Peptide coverage following trypsin + LysC digestion. Green shading indicates peptides identified by MS; white indicates residues not covered.

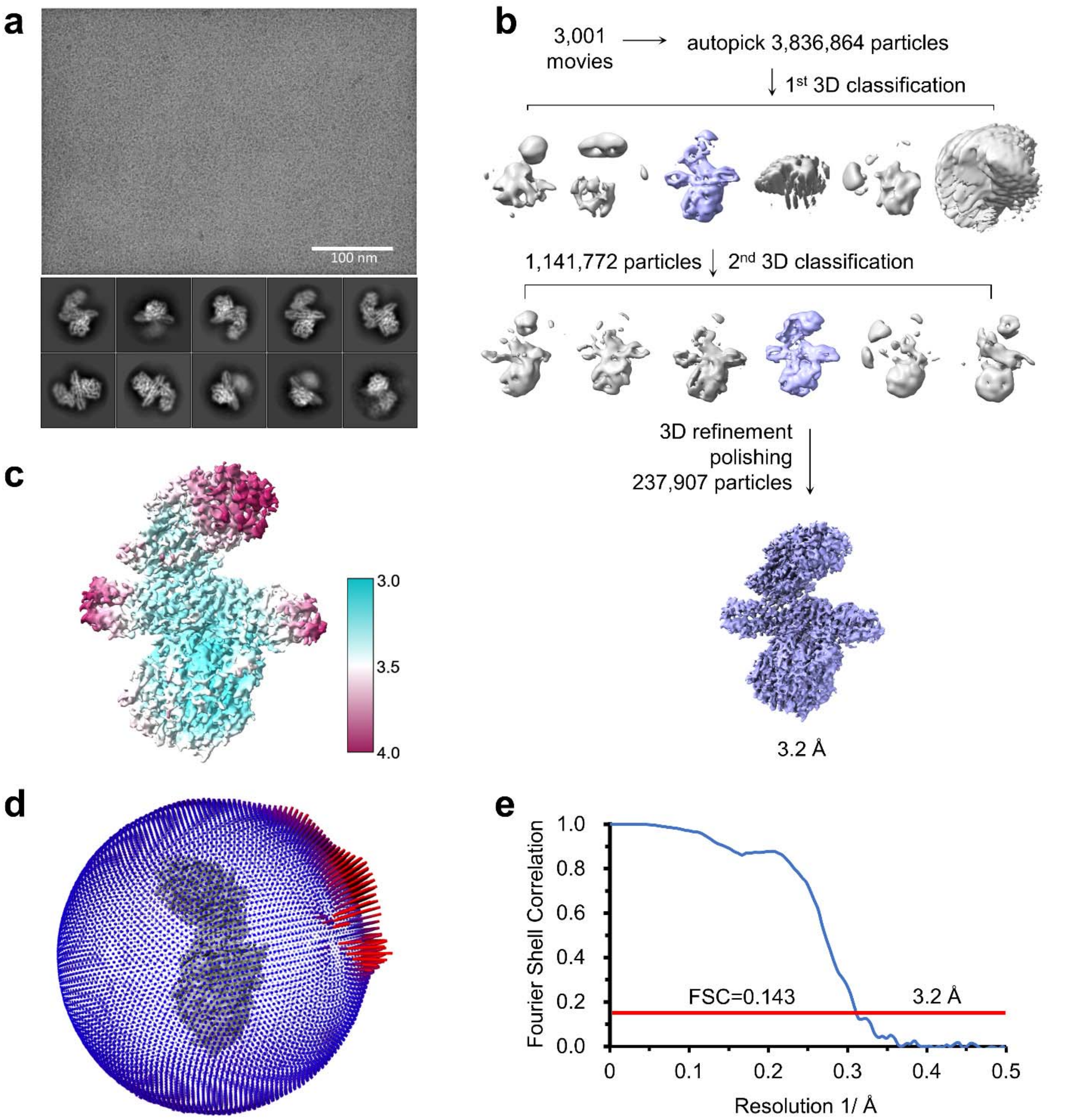

Figure S4 Structure determination of rBoNT/A. (a) Representative cryo-EM micrograph and 2D classification images of rBoNT/A. (b) Flowchart of image processing for rBoNT/A. (c) The density map of rBoNT/A colored by local resolution. (d) Angular distribution plot of particles included in the final 3D reconstruction of rBoNT/A. (e) The Gold standard Fourier Shell Correlation (FSC) curve of the final 3D reconstruction.

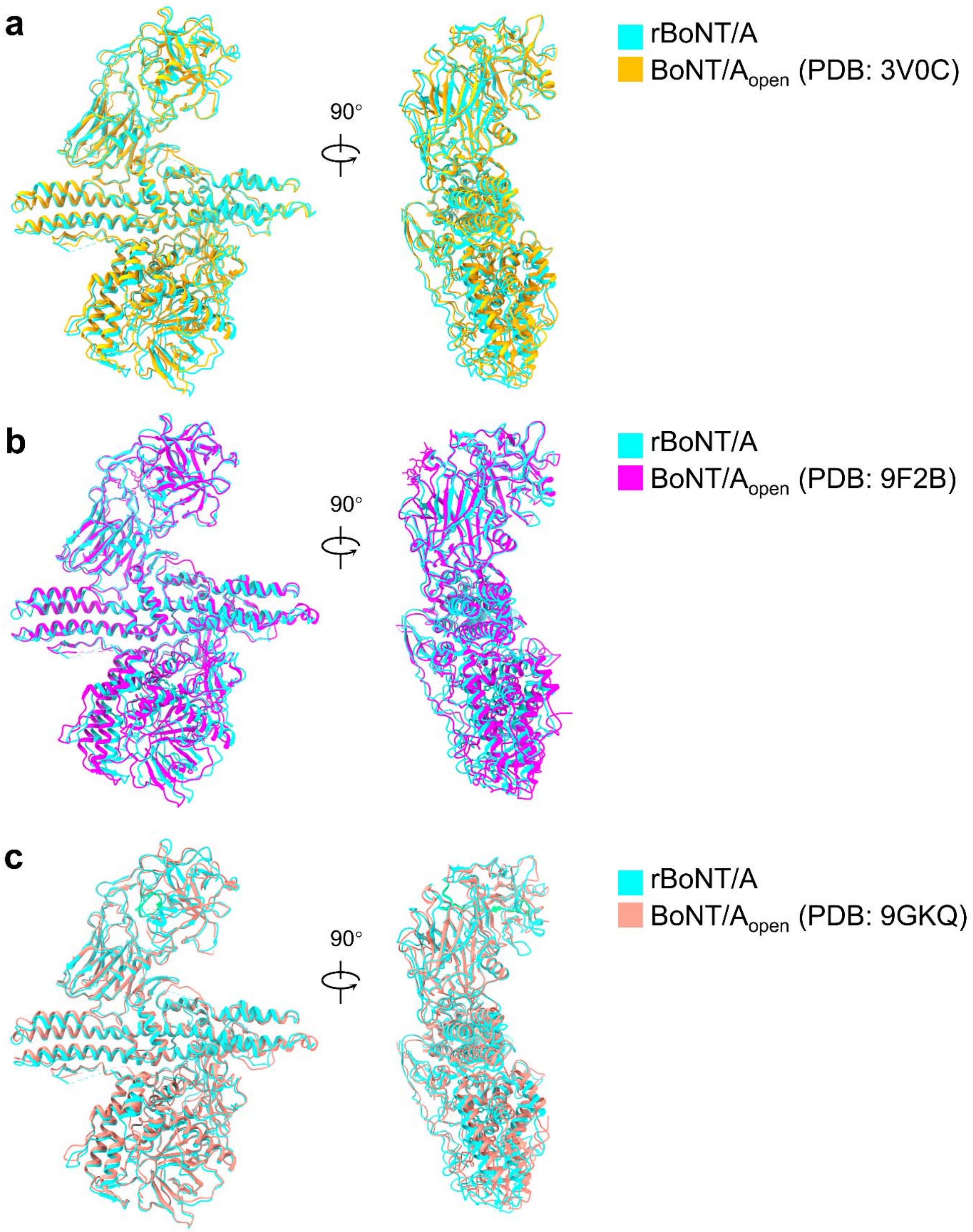

Figure S5 Structural comparison of rBoNT/A with BoNT/A in its open conformations. (a) Side view of the superimposition of rBoNT/A (cyan) and BoNT/A$_{open}$ (PDB: 3V0C, orange) when they are aligned at the whole structure. (b) Side view of the superimposition of rBoNT/A (cyan) and BoNT/A$_{open}$ (PDB:

9F2B, magenta) when they are aligned at the whole structure, and SV2B luminal domain in $BoNT/A_{open}$ (PDB: 9F2B) is omitted for clarity. (c) Side view of the superimposition of rBoNT/A (cyan) and $BoNT/A_{open}$ (PDB: 9GKQ, salmon) when they are aligned at the whole structure.

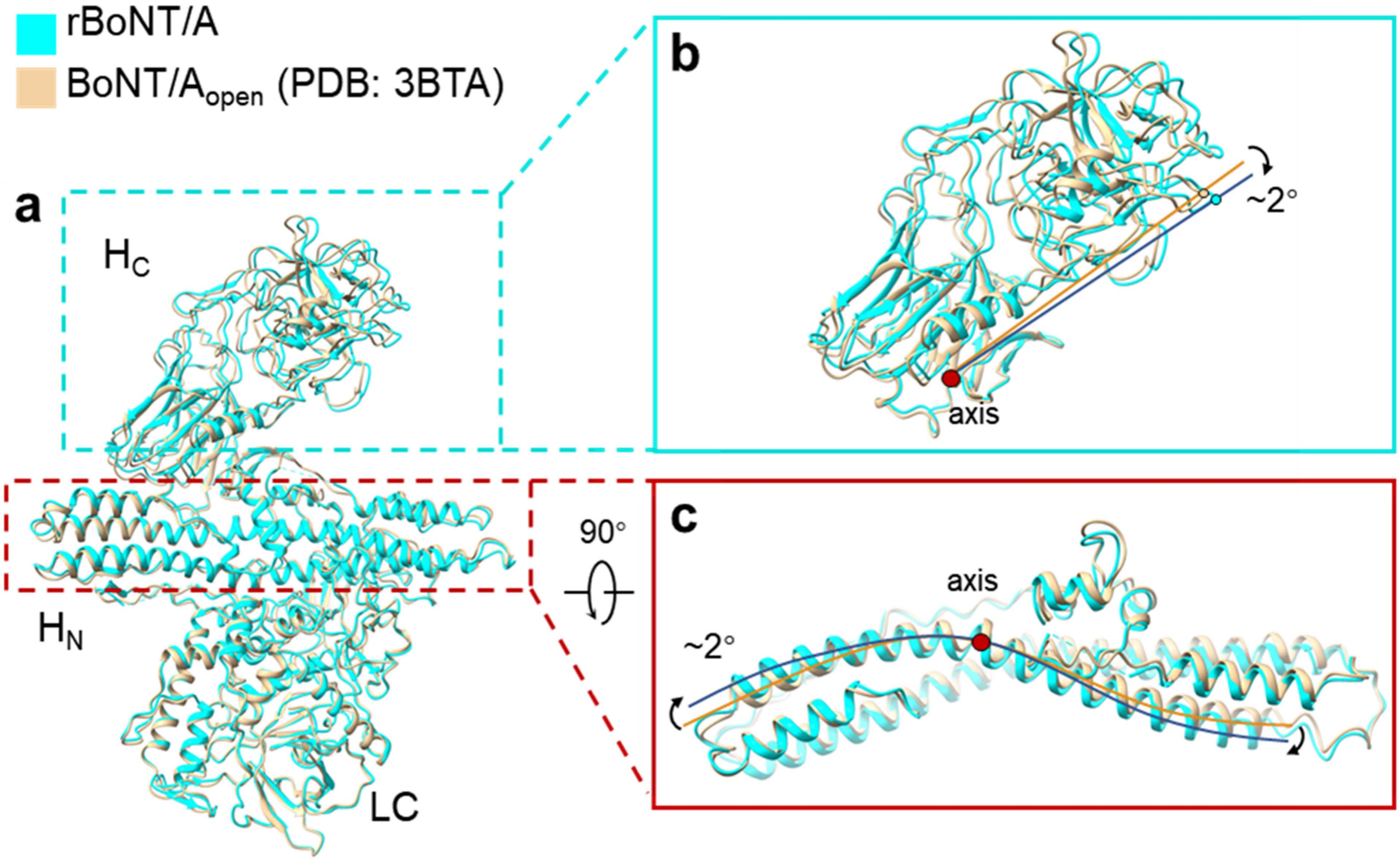

Figure S6 Domain reorientation between native BoNT/A and rBoNT/A. (a) Side view of the superimposition of rBoNT/A (cyan) and native BoNT/A_{open} (PDB: 3BTA, wheat) when they are aligned at the LC. (b) Zoomed-in view of H_C. (c) Zoomed-in view of H_C. Black arrow indicates the rotation of the domain. Red dot indicates the axis.
